# Quantitative Neuromuscular Blockade Monitoring During Surgical Manipulation of the Axillary Artery: A Case Report

**DOI:** 10.1155/cria/6042837

**Published:** 2025-03-23

**Authors:** Valerie K. Yu, Neal W. Fleming

**Affiliations:** Department of Anesthesiology and Pain Medicine, UC Davis Medical Center, Sacramento, California, USA

## Abstract

Vascular surgery often involves the manipulation of major arteries that supply both the central and peripheral nervous systems. Here, we discuss the use of electromyography technology used for neuromuscular blockade monitoring in conjunction with intraoperative neuromonitoring to assess the function of the ulnar nerve during surgical manipulation of the axillary artery. This case highlights the importance of site selection for neuromuscular blockade monitoring as well as the dynamics of the neurovascular system and the resilience that the vasa nervorum may afford peripheral nerves.

## 1. Introduction

Neuromuscular paralysis is often required to optimize surgical conditions and facilitate endotracheal intubation; therefore, intraoperative assessment of neuromuscular blockade (NMB) is an integral part of anesthetic management. Residual blockade has been associated with respiratory complications [1], increased 30-day mortality [1], and delayed postanesthesia care unit discharges [2]; thus, assessment of NMB is essential. As the 2018 International Anesthesia Research Society consensus statement states, quantitative NMB monitoring should be used any time a nondepolarizing NMB drug is administered [3]. Quantitative monitoring is defined as an objective measurement of the train-of-four (TOF) ratio, rather than subjective assessments by visual or tactile means [3]. One of these quantitative monitoring modalities is electromyography (EMG) technology with the TwitchView® monitor (Blink Device Company, Seattle, Washington, United States of America), introduced in July 2018. EMG assesses somatic efferent nerve signals resulting from motor nerve depolarization. Intraoperative neuromonitoring (IONM) also utilizes EMG to monitor intracranial, spinal, and peripheral nerves [4].

For major vascular surgical interventions, in addition to NMB, IONM is used to reduce the incidence of stroke and paraplegia [5]. Procedural manipulation of atherosclerotic vessels and intraoperative hypotension may lead to embolic events or hypoperfusion, which may then result in iatrogenic neurologic injuries [6]. Despite advances in surgical technique, there remains a significant risk of stroke (3.1%–6%) or spinal cord ischemia (2.5%–28%) during aortic surgery, with T4–T8 segments being especially susceptible to decreases in perfusion [6]. For carotid endarterectomy and stenting, the rate of stroke, death, or procedural myocardial infarction has been reported to be 5.2% and 8.5%, respectively [7].

We describe an endovascular surgery case in which the left axillary artery was accessed for the delivery of an endoprosthesis, and during which two modalities, the EMG monitor and IONM, noted a decrease in motor signals in the left upper extremity that recovered to baseline once the axillary artery access site was repaired and closed. The patient provided written Health Insurance Portability and Accountability Act (HIPAA) authorization to publish this case report. This article adheres to the applicable CAse REport (CARE) guidelines.

## 2. Case Presentation

A 70-year-old female (weight: 107 kg and height: 170 cm) with a complex 5.7 cm juxtarenal abdominal aortic aneurysm, presented for endovascular repair using physician-modified endografts. The patient's medical history was significant for mild chronic obstructive pulmonary disease, hypertension, hepatitis C, heart failure with preserved ejection fraction, former smoking (quit 1 month prior), and chronic back pain. The patient was taking hydrochlorothiazide 12.5 mg daily, amlodipine 10 mg daily, carvedilol 12.5 mg daily, and aspirin 81 mg daily. Her anesthetic history included a tubal ligation and an open cholecystectomy. She had also undergone a back surgery without complications, but the spinal levels operated on were unclear to the patient and not specified in her electronic medical record.

In the operating room, standard American Society of Anesthesiologists' monitors were attached, including a 5-lead electrocardiogram, pulse oximeter, noninvasive blood pressure cuff, and end-tidal carbondioxide capnography. A right radial arterial catheter was placed for continuous blood pressure monitoring and blood sampling. EMG monitor electrodes were placed on the left upper extremity to assess the degree of NMB. General anesthesia was induced using lidocaine 100 mg, fentanyl 100 mcg, propofol 100 mg, and rocuronium 100 mg. Total intravenous anesthesia was maintained by propofol and dexmedetomidine infusions to facilitate IONM.

**Figure 1:**
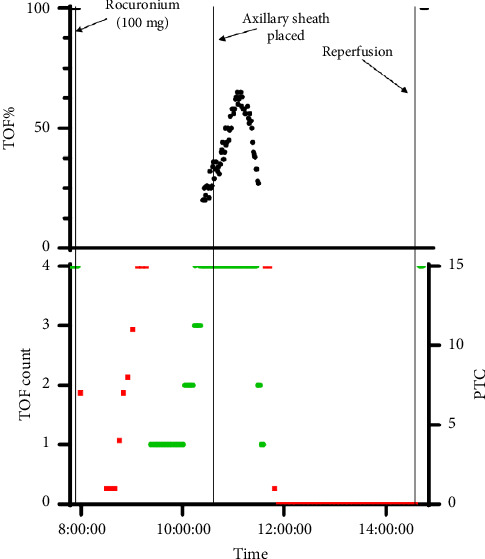
Train-of-Four (TOF) count and TOF ratio assessed by EMG monitor over time. Rocuronium was administered following induction of general anesthesia. The left axillary sheath was placed as indicated. Recovery of muscle activity was rapid and complete almost immediately following reperfusion. TOF count (out of 4 twitches) over time is denoted in green on the left lower axis. Post-tetanic count (PTC, out of 15 counts) is denoted in red on the right lower axis.

Following surgical field preparation, ultrasound-guided access of bilateral common femoral arteries for the delivery of endoprosthesis was obtained; 8Fr 25 cm sheaths were then inserted into each femoral access site, and a pigtail catheter and 0.035″ guidewire were inserted into the right femoral sheath, then advanced to the level of the mid-descending thoracic aorta. Concurrently, open exposure of the left axillary artery for the delivery of an additional endoprosthesis was performed. The axillary artery was identified, 10,000 units of heparin were administered, and an 8Fr sheath was inserted into the axillary artery. The period of complete cross-clamping was brief and thereafter, partial obstruction to flow occurred, but the degree of flow compromise is unknown. At this time, as per quantitative NMB monitoring by the EMG monitor, 4 out of 4 twitches were recovered with the TOF ratio increasing to a peak of 0.65. However, the TOF ratio then began to gradually decrease to a posttetanic count (PTC) of 0 after 47 min. The IONM specialist documented that the motor-evoked potentials (MEPs) and somatosensory-evoked potentials (SSEPs) of the left upper and right lower extremities were decreasing during this same time period. However, motor signals remained fully recovered to baseline in the right upper and left lower extremities. The surgical team was notified of these findings throughout the procedure. PTC monitored by EMG remained at 0 for 2 h and 40 min. Four-vessel fenestrated physician-modified endografts were deployed for this thoracoabdominal aortic aneurysm repair (bilateral renal arteries, the superior mesenteric artery, and the celiac artery were stented). Following the completion of angiogram, all sheaths were removed, and sutures were tied down. Within 5 min following the axillary sheath removal, measurements by the EMG monitor improved from a PTC = 0 to 4 out of 4 twitches measured with a TOF ratio of 1.0. Prior to final incision closure, as per the IONM specialist, MEPs and SSEPs in the left upper and right lower extremities recovered to baseline.  Figure 1 illustrates changes in TOF count and TOF ratio (TOF%) monitored by EMG over the course of the surgery.

The patient did not have any significant intraoperative hypotension and only intermittently required a low-dose phenylephrine infusion running at a rate of 0.05–0.1 mcg·kg^−1^ min^−1^ to maintain a mean arterial pressure greater than 80 mmHg. While further increasing blood pressure is a possible approach to overcome perfusion deficits, in this case, the surgical team felt that increased blood pressure would worsen the risk of bleeding complications and make stent placement more difficult. Given that the axillary sheath was the likely cause of the deficit due to partial obstruction of the flow, the surgical team believed that this deficit would be transient.

We did not administer medications that could potentiate NMB, such as additional rocuronium, magnesium, antibiotics, or sevoflurane. Sugammadex for NMB reversal was not administered because a TOF ratio > 0.9 was measured following reperfusion, as documented by the EMG monitor and confirmed by the IONM specialist. The patient was extubated and transferred to the recovery room with stable vital signs. Two hours postoperation, the recovery registered nurse documented that the patient reported “left hand and finger numbness/tingling.” On exam by the vascular surgery team, bilateral sensation and motors were intact with pulses found with Doppler. The numbness and tingling in her left upper extremity continued to improve. She underwent hourly neurovascular checks in the intensive care unit with a mean arterial pressure goal of > 65 mmHg and a urine output goal of > 50 mL/h. The patient's postoperative intensive care unit course was uncomplicated, and she was transferred from the intensive care unit to wards on postoperative Day #1. She was discharged home 5 days after surgery.

## 3. Discussion

This case demonstrated a strong correlation between quantitative neuromonitoring by EMG technology and findings by IONM. Rocuronium was not redosed after initial administration at induction of general anesthesia, and 2.5 h later, 4 out of 4 twitches were recovered with a TOF ratio of 0.65. Of note, the patient took longer than expected for spontaneous recovery of twitches, 2.5 h after an induction dose of 1 mg/kg, highlighting the importance of intraoperative NMB monitoring. However, following this recovery of twitches, a decrease of twitches in the left upper extremity was noted by the EMG monitor, while IONM also noted a decrease in MEPs and SSEPs from baseline. Concurrently, the surgical team had gained access to the axillary artery with the insertion of an 8Fr sheath. Subsequently, for 2 h and 40 min, the EMG monitor measured 0 out of 4 twitches with PTC = 0, consistent with deep NMB. Yet, the right upper extremity demonstrated full NMB recovery with baseline MEPs and SSEPs as per IONM. Given that twitches had recovered to a TOF ratio of 0.65 prior to this with no subsequent redosing of rocuronium, we suspected that the decrease in signal in the left upper extremity was related to the disruption in perfusion to the ulnar nerve due to surgical manipulation of the left axillary artery. It is also possible that ischemia to the hand muscles themselves contributed to the decrease in signal. Similarly, the IONM findings of decreased MEPs in the right lower extremity were likely also due to a disruption in perfusion given that a pigtail and guidewire were inserted into the right femoral sheath.

Perfusion to peripheral nerves occurs through a continuous longitudinal anastomotic network known as the vasa nervorum that consists of two functionally independent vascular systems [8]. An understanding of the interplay between the vasa nervorum and the peripheral nerve it supplies has many clinical applications in vascular and reconstructive surgery with nerve mobilization and transposition [8]. In this case, the EMG monitor was assessing NMB at the adductor pollicis muscle, which is innervated by the ulnar nerve, which, in turn, is supplied by different arteries. In the axillary section, it is directly supplied by the axillary artery or by a branch of the lateral thoracic artery, while in the upper arm, it is supplied by branches from the collateral ulnar superior artery and in the forearm, by branches of the recurrent ulnar artery [9]. Nerves possess different levels of resilience and vulnerability to ischemia at various sites; their resilience is thought to be due to the use of anaerobic respiration, a high basal blood flow above metabolic requirements, and the dual supply of the vasa nervorum [8]. Much remains to be learned about the vasa nervorum of individual nerves, including the ulnar nerve.

Of note, while the MEPs and SSEPs returned to baseline in the left upper extremity, the patient still experienced transient subjective sensation changes postoperatively, suggesting that there may have been a residual impact from the temporary hypoperfusion of the ulnar nerve, though the specific distribution of symptoms was not documented. Unfortunately, we did not perform a more detailed postoperative examination.

This case is a reminder that surgical interventions may compromise the use of quantitative neuromuscular monitoring such as EMG technology to monitor muscle relaxation. It demonstrates that changes in blood flow can impact neurologic monitors greatly, as well as how resilient peripheral nerves and muscles may be to iatrogenic ischemic insults. In addition, it highlights the importance of selecting the location of NMB monitoring when surgical interventions may significantly impact a particular extremity when IONM may not be readily available to provide additional information.

## Data Availability

Data sharing is not applicable to this article as no new data were created or analyzed in this study.

## Nomenclature

EMG: Electromyography
IONM: Intraoperative neuromonitoring
MEPs: Motor-evoked potentials
NMB: Neuromuscular blockade
PTC: Post-tetanic count
SSEPs: Somatosensory-evoked potentials
TOF: Train-of-four
TOF%: TOF ratio
